# Potential Mechanism Underlying the Role of Mitochondria in Breast Cancer Drug Resistance and Its Related Treatment Prospects

**DOI:** 10.3389/fonc.2021.629614

**Published:** 2021-03-18

**Authors:** Yuefeng Li, Zhian Li

**Affiliations:** Department of Oncological Surgery, Shaoxing Second Hospital, Shaoxing, China

**Keywords:** breast cancer, mitochondrial, drug resistance, chemoresistance, tumor microenvironment

## Abstract

Breast cancer incidence and mortality rates have been consistently high among women. The use of diverse therapeutic strategies, including chemotherapy, endocrine therapy, targeted therapy, and immunotherapy, has improved breast cancer prognosis. However, drug resistance has become a tremendous obstacle in overcoming breast cancer recurrence and metastasis. It is known that mitochondria play an important role in carcinoma cell growth, invasion and apoptosis. Recent studies have explored the involvement of mitochondrial metabolism in breast cancer prognosis. Here, we will provide an overview of studies that investigated mitochondrial metabolism pathways in breast cancer treatment resistance, and discuss the application prospects of agents targeting mitochondrial pathways against drug-resistant breast cancer.

## Introduction

Breast cancer is the second most common cancer in the world and ranks first in cancer incidence in women (1). Its diagnosis rate is increasing year by year, accompanied by a long-term high mortality rate (2). Early breast cancer is usually effectively treated using surgery alone or in combination with adjuvant radiotherapy. However, most patients with advanced breast cancer undergo mastectomy combined with radiotherapy and/or chemotherapy. Notably, the growing popularity of hormone therapy and targeted drug therapy in the treatment of breast cancer has greatly improved its five-year survival rate (3). Although breast cancer treatment methods have progressively diversified, the treatment of breast cancer, especially of triple-negative breast cancer(TNBC), a highly heterogeneous tumor, remains challenging (4), primarily due to chemotherapy resistance (5). Various recent studies have focused on discovering chemotherapy targets and the mechanisms underlying chemotherapy resistance to improve breast cancer prognosis. Furthermore, molecules, such as neuropilin-1 and follistatin-like 1,are considered to be involved in breast cancer resistance to doxorubicin. Nevertheless, the role of these new molecules in specific clinical applications requires further exploration. Therefore, there is an urgent need to explore more effective methods (6, 7).

Recently, the role of mitochondria in cancer has attracted increasing attention. It is well known that mitochondria play an important role in tumor cell occurrence, proliferation and apoptosis. Interestingly, recent studies have shown that tumor chemoresistance is closely related to mitochondria (8). Studies have demonstrated that mitochondrial fission is regulated by dynamin-related protein 1 (Drp-1) and mediated by high-mobility group box 1 protein (HMGB1), a chemotherapy-induced colon cancer product, promoting colorectal cancer tumor chemoresistance (9). Atovaquone, an antiparasitic drug, can effectively block mitochondrial respiration at clinically relevant concentrations, and further increase hepatocellular carcinoma chemosensitivity (10). These studies have laid the foundation for finding the treatments for chemoresistant cancers. In recent years, the role of mitochondria in breast cancer has received substantial attention (11, 12). However, the relationship between mitochondria and chemoresistance in breast cancer has not been systematically examined. This review focuses on studies that investigated the role of mitochondria in breast cancer chemoresistance and discusses the mechanisms and relevant treatment prospects.

### Mitochondrial in Tumor Metabolism and Breast Cancer Drug Resistance

In the human body, tumor cells are found in a dynamic microenvironment, composed of complex stromal cells and extracellular matrix (13), in which mitochondria play an essential role. The original Warburg effect suggests that mitochondrial defects in tumor cells lead to impaired aerobic respiration, which renders tumor cells more prone to aerobic glycolytic metabolism (14, 15). It has been proposed that the changes in tumor cell metabolism are caused by the expression of oncogenes and hypoxia-related signal molecules that upregulate glycolytic enzymes. At the same time hypoxia inducible factor (HIF)-induced pyruvate dehydrogenase kinase (PDK) inhibits the PDH complex, and Akt, an oncogene, mediates the transcription of Glucose transporter type 1 (GLUT1) promoting the binding of hexokinase 2 to the voltage-dependent anion channels (VDAC) on the outer mitochondrial membrane to induce aerobic glycolysis. The synergistic effects of these pathways and the increase of mitochondrial autophagy lead to the glycolytic phenotype of tumor cells (16, 17). On the other hand, a series of studies on the “reverse” Warburg effect revealed that cancer-associated fibroblasts in the tumor microenvironment can change their phenotype through mitochondrial dysfunction and aerobic glycolysis, thereby providing high-energy nutrients to tumor epithelial cells promoting tumor growth, metastasis and chemoresistance (15, 18, 19). Furthermore, Sotgia, F et al. found that 15 molecular markers related to mitochondrial germination and translation were differentially expressed in the tumor microenvironment using an analysis of genome-wide transcription profile data of human breast cancer cells and immunohistochemical verification. In addition, the important role of mitochondria in the metabolic symbiosis between tumor epithelial cancer cells and their surrounding stroma, suggests that the targeting mitochondrial gene expression and translation may be a new treatment approach for breast cancer (20).

**Figure 1:**
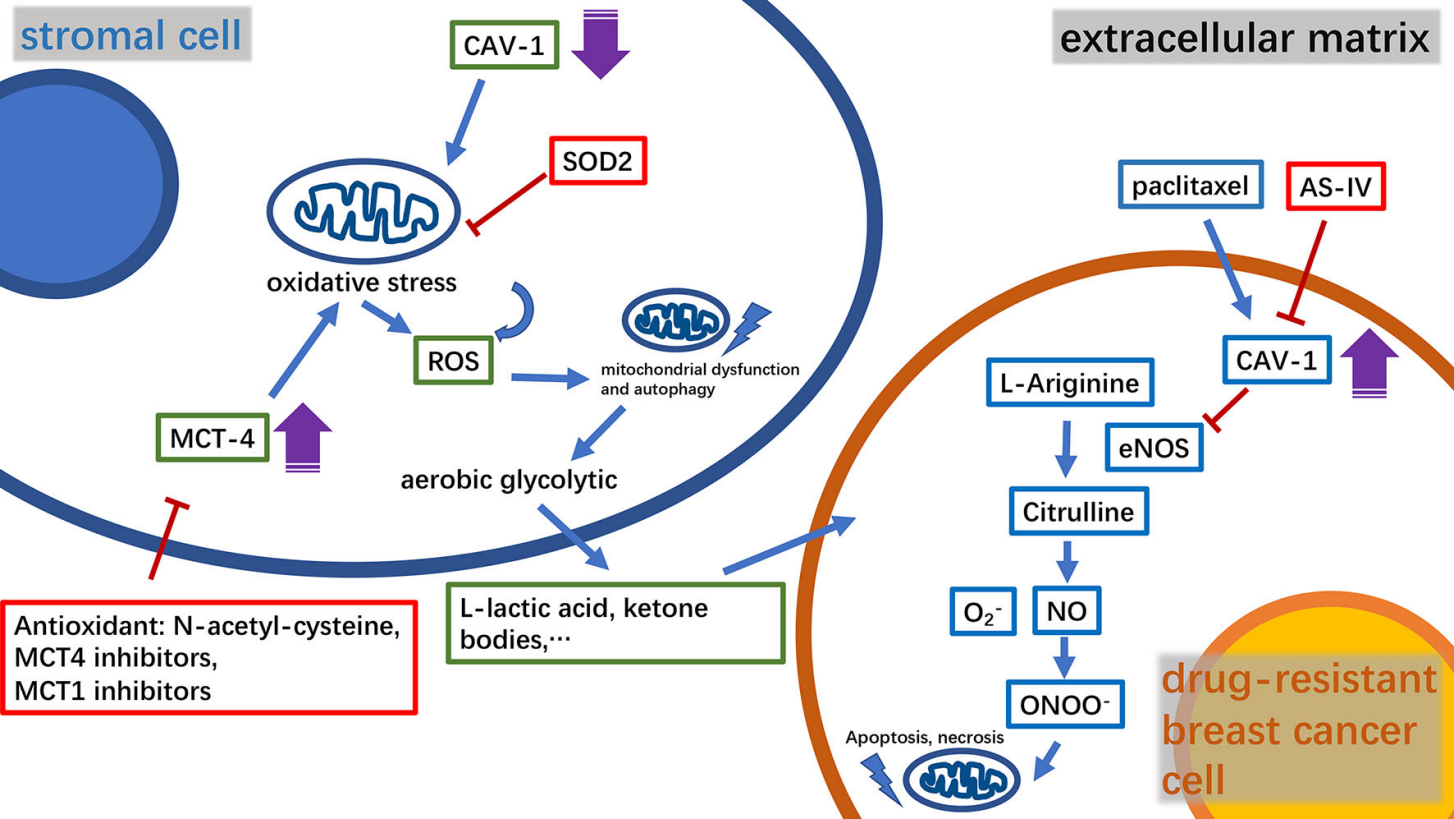
A diagram of the relationship between key molecules in the microenvironment of drug-resistant breast cancer tumors and mitochondria, and the mechanism of the relevant drugs. CAV‐1, caveolin‐1; MCT4, monocarboxylate transporter 4; ROS, reactive oxygen species; SOD2, superoxide dismutase 2; AS‐IV, astragaloside IV; NO, nitric oxide.

The complex metabolic network of the tumor microenvironment is regulated by a variety of molecules, many of which have been found to participate in mitochondria-related metabolic pathways (21, 22). Caveolin-1(CAV-1) is a major structural protein in small plasma membrane invaginations that maintains membrane stability and signal transduction (23). Clinical studies have shown that CAV-1 is an important predictor of breast cancer prognosis (24, 25). Furthermore, it has been found that a decrease in CAV-1 levels in stromal cells in the tumor microenvironment enhances breast cancer resistance to tamoxifen (24). Moreover, in a study using mouse xenograft models, it was found that CAV-1 expression was positively correlated with the tumor sensitivity to nab-paclitaxel (26). CAV-1 downregulation in tumor-associated stromal fibroblasts could increase reactive oxygen species (ROS) production, thus inducing oxidative stress followed by autophagy and mitochondrial dysfunction. The outcome of this metabolic change will promote mitochondrial metabolism using high-energy substances, such as L-lactic acid and ketone bodies to provide nourishment to epithelial cancer cells. The mitochondrial-targeted superoxide dismutase 2(SOD2) is a potential inhibitor that can resist to oxidative stress and block the production of molecules that can nourish tumors, thereby effectively reversing the tumor-promoting phenotype of CAV-1 in breast cancer cells (27). Monocarboxylate transporter 4 (MCT4), an independent prognostic factor for breast cancer survival, was found to be negatively correlated with the expression of CAV-1 (28, 29). Thus, the combined analysis of MCT4 and CAV-1 expression levels in the matrix can improve the accuracy of breast cancer prognosis. The antioxidant N-acetyl-cysteine has been shown to inhibit the oxidative stress-induced formation of MCT4. Furthermore, MCT4 inhibitors can effectively inhibit the influx of L-lactic acid and ketone bodies into the tumor microenvironment, constituting a novel strategy for tumor treatment. However, MCT1 inhibitors are currently considered to have similar efficacy to that of MCT4 and are employed in the clinical studies (29, 30). Corresponding to these findings, CAV-1 was found to be overexpressed in drug-resistant breast cancer cells (31). Astragaloside IV (AS-IV), a biologically active substance purified from Astragalus, can work synergistically with paclitaxel to trigger the mitochondrial apoptosis pathway and effectively induce drug-resistant breast cancer cell death. This process involves AS-IV activation of eNOS/NO/ONOO^−^ signaling by CAV-1 inhibition that enhances the chemosensitivity of breast cancer cells to paclitaxel (32) (**Figure 1**). Intriguingly, the expression level of CAV-1 in TNBC has also been found to be negatively correlated with cancer cell radiation sensitivity (33).

### Mitochondria in Tumor Apoptosis and Breast Cancer Drug Resistance

**Figure 2:**
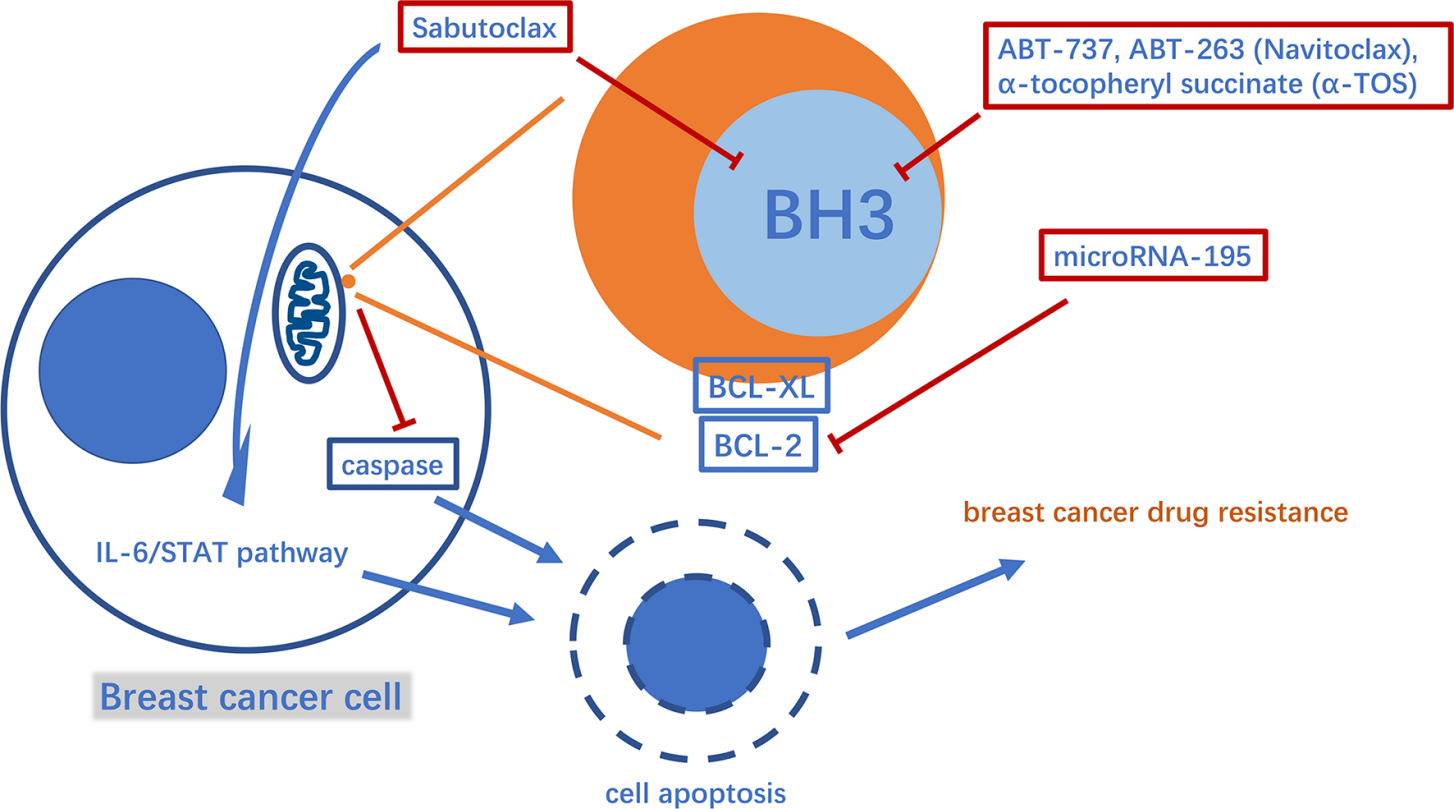
A diagram of some mechanism of mitochondria involved in apoptosis-mediated breast cancer drug resistance and related drugs. BH3, BCL-2 homology domain 3.

B-cell lymphoma 2 (BCL-2) family proteins play a crucial role in the process of regulating cell apoptosis and have both pro-apoptotic and anti-apoptotic activities (34). Typical pro- and anti-apoptotic members are BAX, BCL2, and BCL-XL, respectively (35, 36). Pro-apoptotic proteins such as BAX can promote the release of cytochrome C and second mitochondria-derived activator of caspases (Smac) from mitochondria, leading to cysteinyl aspartate specific proteinase (caspase)-induced cell apoptosis. BCL-2 and BCL-XL inhibit the pro-apoptotic effects of BAX and other molecules (37). In this way, the anti-apoptotic BCL-2 family proteins help breast tumor cells escape apoptosis and acquire drug resistance (38). Thus, inhibiting anti-apoptotic BCL-2 family proteins is a potentially valuable therapeutic strategy against breast cancer drug resistance. Studies have found that microRNA-195 can target BCL-2 to trigger mitochondrial dysfunction and then cause apoptosis, thereby enhancing the therapeutic efficacy of the chemotherapy drug etoposide in breast cancer (39). Via another mechanism, Sabutoclax (BI-97C1), a BCL-2 homology domain 3 (BH3) mimetic, acts as a pan-BCL-2 inhibitor (40). Sabutoclax has shown potent cytotoxicity against drug-resistant breast cancer *in vivo* and *in vitro*. Furthermore, Sabutoclax not only causes the release of caspase from mitochondria to cause cancer cell apoptosis, but also blocks the interleukin 6/signal transducers and activators of transcription (IL-6/STAT) pathway to eliminate the breast cancer stem cells. Interestingly, it has also been successfully used in combination with standard chemotherapy to treat chemoresistant breast cancer (41). Consistent with their principle of action, drugs, such as ABT-737, ABT-263 (Navitoclax) and α-tocopheryl succinate (α-TOS), target BCL-2 in the mitochondria (**Figure 2**). Among them, ABT-737 has been confirmed to improve the docetaxel resistance in TNBC cell lines overexpressing BCL-2 using cytology experiments. Furthermore, using a new technology the drug is encapsulated in poly lactic-co-glycolic acid nanoparticles (NPs) to accumulate drugs in xenograft TNBC tumors and exert effective anti-tumor effects, thus providing a good foundation for successful drug targeting to human breast tumors in the future to avoid systemic side effects (42, 43). It is worth mentioning that Navitoclax has passed phase I of a clinical trial in refractory chronic lymphocytic leukemia, and the optimal drug concentration has also been further explored in a phase II clinical trial. In breast cancer treatment, Navitoclax has been proven to enhance the effectiveness of epidermal growth factor receptor (EGFR)-targeted antibody-drug conjugates for TNBC treatment in animal experiments. In addition, recent studies have also revealed the inherent drug resistance of TNBC to Navitoclax. Therefore, it is necessary to evaluate the specific value of the drug in conjunction with TNBC genomic research (44–46). The combination of α-TOS and high-dose tamoxifen can effectively inhibit the proliferation activity of TNBC, and improve the anti-cancer effect of pterostilbene in breast cancer xenograft mice. In a recent study, pluronic polymer (P123) was modified into ortho ester End-capping (P123-OE) and bridged with a-TOS to form a copolymer (POT), and then doxorubicin-loaded POT micelles (POT-DOX) were used in breast cancer animal models, which effectively increased the accumulation of drugs in multi-drug-resistant breast cancer cells and enhanced the drug’s anti-cancer effects, potentially providing alternative clinical treatment options (47–49). Myeloid cell leukemia-1 (MCL1) is another typical anti-apoptotic protein belonging to the BCL-2 family (50). The myelocytomatosis oncogene (*MYC*), a proto-oncogene, encodes a transcription factor involved in cancer cell proliferation and apoptosis (51, 52). The mRNA and protein levels of these two molecules were co-amplified in paclitaxel-resistant breast cancer cell lines. MYC and MCL-1 mediate the enrichment of breast cancer stem cells(CSCs) (53, 54). Among them, MCL2 has also been confirmed to be located in the mitochondrial matrix to enhance mitochondrial oxidative phosphorylation(mtOXPHOS), which in turn increases the ROS generation, activates hypoxia stress, and causes drug resistance-mediated CSC enrichment (53, 55). Therefore, drugs that inhibit HIF-1a, such as N-acetylcysteine, oligomycin and digoxin, may provide direction in the treatment of drug-resistant breast cancer (56–58).

### Mitochondrial Dynamics and Breast Cancer Drug Resistance

Mitochondria are highly dynamic organelles that change their shape, size, and distribution to adapt to changes in different cell states *via* the coordinate action of fission and fusion (59). Dynamin-related protein 1 (Drp1), a classical mitochondrial fission protein, can cause mitochondria to divide into two to form a small circular mitochondrial fragment network. Mitochondrial fusion protein (MFN) and optic atrophy 1(OPA1), can elongate or cluster together through fusion (59–61). A study of tamoxifen-resistant breast cell lines found that the drug-resistant cell lines have a more fragmented mitochondrial network. In these cell lines, serine 637, an essential phosphorylation site of DRP1, was activated, while serine 616, another vital phosphorylation site, was not, which increased the mitochondrial fission activity of DRP1, causing mitochondrial fragmentation. Based on this, changes in mitochondrial dynamics that are closely related to breast cancer drug-resistance can be improved (62). Current research on drugs targeting mitochondrial dynamics is mostly focused on breast cancer growth and proliferation rather than breast cancer drug resistance (63–65). Mitochondrial transplantation is a new type of biological technology that gradually extends from animal models to human clinical applications (66). Using this technology, exogenous healthy mitochondria are transplanted into cells with damaged mitochondria to achieve the treatment purpose (67). A study on mitochondrial transplantation in breast cancer cell lines found that the protein levels of MFN2 and OPA1 in the cells significantly increased after the transplantation of exogenous healthy mitochondria into breast cancer cells, while the protein level of drp1 dramatically decreased. Interestingly, the morphology of the mitochondria in the cell was mostly elongated to tubular, while the fragmented mitochondria were obviously inhibited. In addition, the resistance of breast cancer cells to the anticancer drugs doxorubicin and paclitaxel was also significantly reduced (68). Recent studies have also suggested that mitochondrial transplantation can change the tumor microenvironment to combat breast cancer, and demonstrated the therapeutic effect of mitochondrial transplantation on breast cancer in animal models (69). In these studies, we observed that while mitochondrial transplantation brings about changes in mitochondrial morphology, it also regulates functions, such as oxidative respiration. The morphology and function of mitochondria are inextricably linked (68, 69). Mitochondrial transplantation is a new approach for the treatment of drug-resistant breast cancer.

### Mitochondrial DNA and Breast Cancer Drug-Resistance

Mitochondrial DNA(mtDNA), a double-stranded circular DNA, contains 37 genes encoding rRNA, tRNA, and oxidative phosphorylation complex-related proteins (70). A variety of treatments that target mitochondrial gene expression have been explored in a variety of diseases, including breast cancer, with proven therapeutic benefits (71). The link between mitochondrial gene copy number and breast cancer treatment resistance has attracted increasing attention (72, 73). Metformin can stunt breast cancer progress by inhibiting complex I encoded by the electron transport chain gene in mtDNA (74, 75). The decrease in BTB and CNC homology 1(BACH1), a hemin-binding transcription factor, can promote the expression of the electron transport chain(ETC) genes to increase the sensitivity of breast cancer to metformin. The specific degradation of BACH1 by panhematin (an FDA-approved drug) can effectively enhance the sensitivity of breast cancer cells to metformin treatment *in vitro* and *in vivo* (76). Not long ago, another extraordinary study found that cancer-related fibroblast-derived exosomes with complete genomic mitochondrial DNA can be obtained by breast stem cell-like cancer cells and display mtOXPHOS-dependent breast cancer endocrine-resistant therapy (77). Hitherto, drugs that target mtDNA, such as vitamin K3 (menadione), are often used in combination with other drugs in the treatment of breast cancer (78, 79). However, whether they improve resistance to breast cancer treatment remains unclear. This is worth exploring in future studies.

## Conclusions

The problem of drug resistance in the comprehensive treatment model of breast cancer has been extensively investigated, and mitochondria have been found to play a subtle role in the process of breast cancer drug resistance. Exploration of the role of mitochondria in breast cancer drug resistance in the tumor microenvironment and cancer cell interior indicates that: 1) numerous molecules in the tumor microenvironment can mediate the production of a variety of metabolites to induce drug resistance through the action of mitochondria, 2) mitochondria can regulate cell apoptosis and affect breast cancer resistance, 3) morphological and functional changes in the mitochondria can promote breast cancer resistance, and 4) the expression level of mtDNA can mediate breast cancer resistance. These studies have proposed the use of effective molecular targeted drugs or new treatments to sensitize breast cancer cells to drugs, and some drugs are already used in clinical research. Exploring the role of mitochondria in breast cancer chemoresistance is expected to open up novel ways for breast cancer treatment.

## Author Contributions

YL: literature search, writing, and editing of the manuscript. ZL: Analysis, design, writing-review and edit, and supervision. All authors contributed to the article and approved the submitted version.

## Conflict of Interest

The authors declare that the research was conducted in the absence of any commercial or financial relationships that could be construed as a potential conflict of interest.
